# Caroli Disease: A Presentation of Acute Pancreatitis and Cholangitis

**DOI:** 10.7759/cureus.9135

**Published:** 2020-07-11

**Authors:** Muhammad Z Khan, Asim Kichloo, Zain El-Amir, Muhammad Shah zaib, Farah Wani

**Affiliations:** 1 Internal Medicine, Central Michigan University College of Medicine, Saginaw, USA; 2 Internal Medicine, Central Michigan University, Saginaw, USA; 3 Internal Medicine, Nishtar Medical University and Hospital, Multan, PAK; 4 Family Medicine, Samaritan Medical Center, Watertown, USA

**Keywords:** caroli disease, congenital, pancreatitis, cholangitis, ercp, ct, mrcp, transplant, cystic disease, ursodeoxycholic acid

## Abstract

Caroli disease is a rare congenital disorder resulting from the dilation of large intrahepatic bile ducts. Patients affected with Caroli disease are at increased risk of complications resulting from bile stasis and stone formation. We report the case of a 37-year-old woman with a past surgical history of cholecystectomy who presented to the emergency room with a chief complaint of abdominal pain and nausea. The pain was characteristic of acute pancreatitis but she was hemodynamically stable. Total bilirubin was 4.1 mg/dL with a direct fraction of 3.1 mg/dL, aspartate aminotransferase (AST) and alanine aminotransferase (ALT) were 850 IU/L and 1025 IU/L, respectively. Serum amylase and lipase were elevated at 581 IU/L and 1328 IU/L, respectively. CT scan of abdomen/pelvis without contrast showed common bile duct (CBD) measuring 1.6 cm with intrahepatic biliary system dilation and mild peripancreatic fat stranding. She was diagnosed with acute pancreatitis. On the second day, she developed a temperature of 99.6°F. Hepatitis immunity panel was negative for acute hepatitis. The patient was started on antibiotics (IV ciprofloxacin and metronidazole) for suspicion of acute cholangitis. Endoscopic retrograde cholangiopancreatography (ERCP) was done which showed mild dilated intrahepatic ducts and CBD dilation of 1.6 cm, and a choledochal cyst at CBD. Sphincterotomy was done and good bile drainage was reported. She was later discharged in a stable condition. Caroli disease affects males and females equally and most are diagnosed before the age of 30 years correlated with the onset of symptoms. By far, the most commonly reported symptom is acute cholangitis but pancreatitis occurs rarely. Recurrent bouts of infection lead to portal hypertension, fibrosis of the liver and ultimately end up with an orthotopic liver transplant (OLT). Regular follow-ups are important for disease surveillance and monitoring.

## Introduction

Caroli disease is a rare congenital disorder with a prevalence of about one in 1,000,000 [1]. Two types have been described so far which result from biliary ductal dilatation. Caroli disease or simply bile ductal ectasia and Caroli syndrome, which is more common and associated with congenital hepatic fibrosis [2,3]. Another classification system includes choledochal cyst type V as a form of Caroli disease [3,4]. The affected patients are at increased risk of complications resulting from bile stasis and stone formation. Other complications include recurrent bouts of cholangitis and pancreatitis which is a cause of significant morbidity in these patients [2]. The case described highlights the presenting features of the disease and the common diagnostic and therapeutic interventions.

## Case presentation

A 37-year-old woman, with a history of cholecystectomy due to recurrent biliary colic, came to the emergency room with complaints of abdominal pain and nausea for the last two days. The pain was in the epigastric region, 10/10 in intensity and cramping with radiation to the back. At the time of admission, the patient was hemodynamically stable with positive findings of epigastric tenderness on deep palpation. Blood pressure was 118/85 mmHg, heart rate of 88 beats per min and temperature of 97.6°F. Total bilirubin was 4.1 mg/dL with a direct fraction of 3.1 mg/dL, aspartate aminotransferase (AST) and alanine aminotransferase (ALT) were 850 IU/L and 1025 IU/L, respectively. Serum amylase and lipase were elevated at 581 IU/L and 1328 IU/L, respectively. The patient was admitted under the impression of acute pancreatitis with Bedside Index of Severity in Acute Pancreatitis (BISAP) score of zero, Ranson’s Score on admission was one. CT scan of abdomen/pelvis without contrast showed CBD measuring 1.6 cm with intrahepatic biliary system dilation and mild peripancreatic fat stranding. On the second day, she developed a temperature of 99.6°F. Hepatitis immunity panel was negative for acute hepatitis. Patient was started on antibiotics (IV ciprofloxacin and metronidazole) for suspicion of acute cholangitis. Endoscopic retrograde cholangiopancreatography (ERCP) was done which showed mild dilated intrahepatic ducts; possible variant of Caroli disease, CBD dilation of 1.6 cm, and a choledochal cyst at CBD. Sphincterotomy was done and good bile drainage was reported. Post-procedure magnetic resonance cholangiopancreatography (MRCP) showed mild intra- and extra-hepatic biliary dilation. No focal filling defect was seen in the biliary tree. Post-ERCP, the patient improved clinically and subjectively. Laboratory values showed a downward trend and the patient was discharged on oral antibiotics.

## Discussion

Caroli disease affects males and females equally and most are diagnosed before the age of 30 correlated with the onset of symptoms [5]. Both autosomal dominant and recessive modes of inheritance are common and the association with the cystic anomalies of the kidney is also reported. The association with congenital hepatic fibrosis (CHF) is commonly referred to as Caroli’s syndrome which has been described above [5].

By far, the most commonly reported symptom is acute cholangitis and the patients present with the classic triad of jaundice, right upper pain and fever [6]. Recurrent bouts of infection lead to portal hypertension, fibrosis of the liver and ultimately end up with orthotopic liver transplant (OLT) [5]. A study conducted in the large United States Healthcare center showed that 96 out of 104 patients with Caroli disease had a liver transplant procedure and the rest of the eight had a combined liver/kidney transplant [7]. This illustrates the importance of polycystic kidney disease that often occurs concurrently with a percentage as high as 60%-80% [7]. Bile stasis resulting from ductal ectasia predisposes to stone formation. The molecular analysis usually puts the cholesterol stones as the most frequently encountered type [8]. Ursodeoxycholic acid (UDCA) has been shown to result in the dissolution and subsequent improvement in symptoms resulting from hepatolithiasis [3,9]. Altered ductal anatomy from dilatation and choledochal cysts in conjunction with stone formation can sometimes obstruct the pancreatic duct, which is rare; that’s the reason for the low incidence of pancreatitis reported in Caroli disease [2,9]. Although the exact numbers are not available for the incidence of this finding, a few case reports of pancreatitis in association with either Caroli disease or cavernous ectasia of the pancreatic ducts have been found in the literature. A 23-year-old male with a history of pancreatic ductal cysts had repeated attacks of pancreatitis resulting in significant morbidity [10]. Similarly, a child was diagnosed with Caroli disease based on recurrent pancreatitis not amenable to the commonly employed treatment strategies [11].

**Figure 1:**
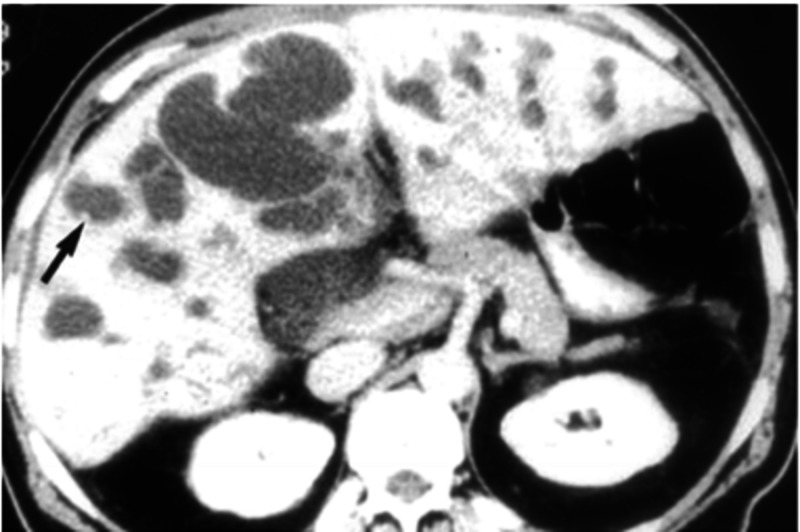
Contrast-enhanced CT scan Contrast-enhanced CT showing intrahepatic and extrahepatic bile duct dilations with a black arrow pointing out fibrovascular bundles. Photo from Levy AD, Rohrmann CA, Murakata LA, Lonergan GJ. Caroli's Disease: Radiologic Spectrum with Pathologic Correlation. American Journal of Roentgenology.

With the advances made in radiologic and endoscopic procedures, Caroli disease is now being diagnosed with far greater accuracy and precision than it used to be in the past. Ultrasound (US) and contrast-enhanced CT scan are employed as the first-line diagnostic modalities [12,13]. In our patient here, CT scan without contrast revealed dilation of the CBD and intrahepatic biliary tree. This is consistent with the saccular and fusiform dilations of the intrahepatic biliary tree on CT scans of Caroli disease patients (Figure 1).

 

Once initial imaging has been done, ERCP is performed as a therapeutic tool which also aids in better visualization of the ductal malformation and choledochal cysts, if present [6]. The aforementioned techniques were employed in the care of our patient and proved to be invaluable in the diagnosis and management.

Treatment for Caroli disease is centered around the management of symptoms; antibiotics for cholangitis, UDCA for hepatolithiasis, and treating pancreatitis accordingly. Complications include cholangiocarcinoma and hepatic fibrosis leading to decompensated liver disease. Liver resection is another surgical option available and is a subject of the ongoing debate between clinicians. As the disease affects the hepatobiliary system, patients should have regular follow up which includes the abdominal US, markers of inflammation like ALT, AST, C-reactive protein (CRP) and serial blood counts.

## Conclusions

Caroli disease and syndrome are rare and overlapping entities. The patients might go undiagnosed for a long time, but an attack of pancreatitis or cholangitis brings them to the attention of the examining physician. It is important to improve the quality of life by addressing the symptoms. Cholangiocarcinoma and hepatic fibrosis are some of the complications for which regular follow-ups are recommended. The case presented here is a good example of the way patients present and what diagnostic and treatment strategies should be employed for the management.
